# Behavioural Parameters of Circadian Rhythm Are Not Correlated with Dim Light Melatonin Onset: An Observational Study on Healthy Volunteers

**DOI:** 10.3390/jcm12247757

**Published:** 2023-12-18

**Authors:** Michał Mateusz Dermanowski, Adam Wichniak, Arkadiusz Hejduk, Julita Kuczyńska, Monika Dominiak, Paweł Mierzejewski

**Affiliations:** 1 Department of Pharmacology, Institute of Psychiatry and Neurology, Sobieskiego 9, 02-957 Warsaw, Poland; mdermanowski@ipin.edu.pl (M.M.D.); jkuczynska@ipin.edu.pl (J.K.); mdominiak@ipin.edu.pl (M.D.); 2 Third Department of Psychiatry, Institute of Psychiatry and Neurology, Sobieskiego 9, 02-957 Warsaw, Poland; wichniak@ipin.edu.pl; 3 Department of Research and Development, LEK-AM Pharmaceutical Company Ltd., Ostrzykowizna 14A, 05-170 Zakroczym, Poland; arkadiuszhejduk@lekam.pl

**Keywords:** dim light melatonin onset, melatonin, actigraphy, sleep diary, insomnia, sleepiness, chronotype, circadian rhythm

## Abstract

Dim light melatonin onset (DLMO) is considered the most reliable marker of the circadian rhythm phase in humans. DLMO may moderately correlate with sleep onset and sleep offset time. There are no sufficient data about the correlations between DLMO and clinical scales assessing sleep quality and daytime symptoms of poor night sleep. The aim of the study was to determine the association between DLMO and basic sleep parameters from actigraphy and sleep diaries, as well as the association between DLMO and the following insomnia clinical scales: the Athens Insomnia Scale (AIS), Insomnia Severity Index (ISI), Epworth Sleepiness Scale (ESS), and chronotype questionnaires: Morningness–Eveningness Questionnaire (MEQ) and Composite Scale of Morningness (CSM). Participants of the study were healthy volunteers. Sleep parameters were measured by sleep diaries and actigraphy, and the following clinical scales: the AIS, ISI, and ESS, and chronotype questionnaires: MEQ and CSM. DLMO was calculated based on plasma melatonin concentration. The blood samples were collected hourly at five time points between 20:00 and 00:00 during the session in dim red light (<50 lux). Melatonin concertation was determined by LC-MS/MS. Twenty-one volunteers participated in the study. DLMO was calculated in 12 participants. There was a significant correlation between DLMO and ISI (r = 0.60, *p* = 0.038) and ESS (r = 0.61, *p* = 0.034). The correlation coefficient between the DLMO and the AIS was also high, however insignificant (r = 0.57, *p* = 0.054). There were no significant correlations between DLMO and chronotype scales MEQ and CSM. DLMO did not correlate with sleep onset and sleep offset; however, DLMO correlated with the Sleep Fragmentation Index (SFI) (r = 0.67, *p* = 0.017). DLMO is associated with poorer sleep maintenance, a stronger feeling of insomnia, and sleepiness during the day. Simultaneously, chronotype pattern and circadian rhythm parameters do not correlate with DLMO. Biological circadian rhythm does not reflect the real-life sleep–wake rhythm, indicating that the lifestyle is more often disconnected from the biological clock.

## 1. Introduction

Melatonin is a neurohormone produced in the pineal gland that regulates circadian rhythmicity. In healthy individuals, melatonin secretion begins in the evening and reaches its peak at about 2–4 a.m. The time during the evening when melatonin concentrations begin to rise is called dim light melatonin onset (DLMO). DLMO is determined by measuring melatonin concentration during the evening and night hours. DLMO can be determined from both plasma and saliva samples [1]. The melatonin concentration in saliva and plasma, as well as DLMO, is highly correlated at r = 0.76, and r = 0.69, respectively. According to our previous study in healthy adults, the average DLMO time was 21:30 ± 0:45 [1], which was similar to the results of other studies [2,3,4,5].

DLMO usually occurs 2–3 h before the onset of sleep [6] and is considered the reliable marker of the circadian rhythm phase in humans [7,8]. In people with insomnia, DLMO occurs later, which also raises the interest of this marker in sleep disorders [9,10,11]. It has been proposed that DLMO should be measured before treatments of circadian rhythm disorders [12].

The aim of the present study was to determine the association between DLMO and basic sleep parameters from actigraphy and sleep diaries, as well as the association between DLMO and the following commonly used insomnia clinical scales: the Athens Insomnia Scale (AIS), Insomnia Severity Index (ISI), Epworth Sleepiness Scale (ESS), and chronotype questionnaires: Morningness–Eveningness Questionnaire (MEQ) and Composite Scale of Morningness (CSM).

Previous studies indicate a moderate correlation between DLMO and circadian rhythm as measured by the MEQ [13]. Based on this analysis, it can be concluded that it is not clear how DLMO correlates with the MEQ scores, with many studies finding no correlation. CSM was an attempt to extract the best items from other sleep questionnaires [14]. Both MEQ and CSM have high levels of reliability (>0.80) [15]. We decided to check the correlation between DLMO and the CSM. Until now, no one has studied this on healthy volunteers.

Circadian rhythm disorders, especially delayed circadian rhythms, can be a significant factor in sleep problems [16]. To date, no one has investigated the correlation between DLMO and the Athens Insomnia Scale (AIS). A study conducted on shift workers found a moderate, negative correlation between DLMO and the Insomnia Severity Index (ISI) where r = −0.68 and the Epworth Sleepiness Scale (ESS) where r = −0.55 [17]. In our study, we decided to verify the above observations.

Only single studies analyzed the relationship between DLMO and actigraphy parameters, showing, among others, a negative correlation between DLMO and light exposure (r = −0.35) or activity (r = −0.33) [18]. Actigraphy, like DLMO, is an objective continuous method of measuring activity throughout the day; therefore, it was expected that there would be significant relationships between the parameters obtained by actigraphy and DLMO.

A more subjective, but easier to apply, method of analyzing sleep parameters is a sleep diary. Studies on young adults indicate a strong correlation between DLMO and the midpoint of sleep (r = 0.89, r = 0.68); however, a weaker correlation was found for bedtime (r = 0.36) [19,20]. On the other hand, in a study on patients suffering from Delayed Sleep Wake Phase Disorder (DSWPD), the correlations between DLMO and sleep parameters (sleep onset, r = 0.22, midsleep, r = 0.16, wake up time, r = −0.04) were weak and not statistically significant [21]. Studies in which significant correlations were observed were usually conducted on young adults or adolescents, and it is difficult to relate them to the older population. Some studies indicate that DLMO may correlate moderately with sleep onset (r = 0.47–0.77 and r = 0.43–0.53, based on sleep diary and actigraphy, respectively) [20,21,22,23,24,25], sleep offset (r = 0.44–0.79 and r = 0.57–0.66 based on sleep diary and actigraphy, respectively) [20,21,23,24], bedtime (r = 0.60 based on sleep diary) [22], and out-of-bed time (r = 0.58 based on sleep diary) [21].

It should be highlighted that studies on the relationship between DLMO or sleep parameters derived from the sleep diary and actigraphy are not fully consistent and coherent. One may notice that the results regarding these correlations vary considerably between studies. It should be emphasized that these were conducted on very demographically and clinically diverse populations. In regard to the correlations of DLMO with clinical scales assessing sleep quality and daytime symptoms of poor night sleep, such studies are utterly lacking. Hence, the aim of this study was to analyze the correlation between DLMO with a whole battery of basic sleep parameters from actigraphy and sleep diaries, and the most important clinical scales: the Athens Insomnia Scale (AIS), Insomnia Severity Index (ISI), and Epworth Sleepiness Scale (ESS), as well as chronotype questionnaires: Morningness–Eveningness Questionnaire (MEQ) and Composite Scale of Morningness (CSM) on a healthy middle-aged adult population.

## 2. Materials and Methods

### 2.1. Study Group

The study was conducted at the Sleep Medicine Center and at the Department of Pharmacology at the Institute of Psychiatry and Neurology in Warsaw, Poland. The study protocol was approved by the Local Medical Ethics Committee (No. 23/2018). Volunteers were recruited from among the employees of the Institute of Psychiatry and Neurology. All participants gave written informed consent. Determination of plasma melatonin levels was performed on a group of 21 healthy volunteers.

Inclusion criteria were age 18–65, both sexes, with a typical sleep phase (usual bedtime between 22:00 and 01:00), who were capable of giving informed consent and gave written informed consent to participate in the study. Exclusion criteria were diseases that could affect melatonin secretion/metabolism (untreated hormonal/metabolic/liver diseases, chronic inflammatory/autoimmune diseases, and sleep disorders), taking drugs that interact with melatonin (beta-blockers, antidepressants, and glucocorticoids) [25,26], blood clotting disorders, alcohol consumption within 48 h of the study session, having an on-call or night shift within 7 days of the study session, and time zone change within 30 days of the study session.

### 2.2. Study Session

The study was conducted between December 2018 and February 2019. Sample collections for melatonin measurements were conducted during three sessions, each with 7 participants, under low red light conditions (<50 lux) from 19:00 to 00:00. Volunteers were prohibited from consuming caffeine-containing beverages and smoking for the entire study session (for details see Dermanowski et al. 2022) [1]. Participants slept at their habitual times with no restrictions on their sleep schedules.

The whole blood was collected at five time points: 20:00, 21:00, 22:00, 23:00, and 00:00, into tubes with K_3_EDTA anticoagulant (S-Monovette^®^, Sarstedt, Nümbrecht, Germany). Blood samples were centrifuged for 10 min at 1000× *g*. Received plasma samples were frozen at −60 °C until analysis.

### 2.3. Clinical Scales and Questionnaires

Each participant was asked to complete the following clinical scales: the Athens Insomnia Scale (AIS) [27,28], Insomnia Severity Index (ISI) [29], Epworth Sleepiness Scale (ESS) [30], and chronotype questionnaires: the Morningness–Eveningness Questionnaire (MEQ) [31,32] and the Composite Scale of Morningness (CSM) [14,33]. All scales were completed by participants at home before melatonin examination.

The AIS is used to assess the presence of insomnia. An AIS score ≥8 indicates the presence of insomnia symptoms. Another insomnia scale frequently used is ISI. As in the case of AIS, the score ≥8 indicates the presence of insomnia symptoms. The reliability of AIS is good with a mean Cronbach’s alpha of 0.86, mean item-total correlation range of 0.56–0.80, and mean intraclass correlation coefficient (ICC) at different time intervals of 0.78 [34]. ISI is based on diagnostic criteria according to the Diagnostic and Statistical Manual of Mental Disorders [29]. The reliability of the ISI is good, with a mean Cronbach’s alpha coefficient of 0.82, mean adjusted item-total correlations ranging from 0.47 to 0.66 and good test–retest reliability at 2 weeks (mean ICC = 0.82) [34]. The ESS is used to assess the occurrence and severity of daytime sleepiness, which may be related to insufficient sleep at night. An ESS score ≥11 indicates excessive daytime sleepiness. The confirmed reliability of the ESS averages of Cronbach’s alpha of 0.82, and the mean item-total correlations range from 0.38 to 0.69. The internal consistency of the questionnaire is also confirmed through test–retest reliability analysis with an interval of 7 to 35 days (mean ICC = 0.84) [30].

The MEQ is used to assess chronotype. An MEQ score of 16–41 indicates an evening chronotype, 42–58 represents an intermediate chronotype, and 59–87 represents a morning chronotype. Another chronotype questionnaire used to assess chronotype is CSM. A CSM score of 0–24 indicates an evening chronotype, 24–42 indicates an intermediate chronotype, and 43–55 indicates a morning chronotype. In regard to the reliability of the MEQ, studies consistently show it to be high (>0.80) [15]. The reliability of the CSM is high (Cronbach’s alpha = 0.87) [35]. The test–retest reliability at 11 weeks is also good = 0.89 [36].

### 2.4. Sleep Diary and Actigraphy

Prior to the laboratory study session, participants were given sleep diaries, which they completed over a period of at least seven days. The sleep diary included time of going to bed, time of lights out (lights-out time), latency to lights out, sleep latency, time of falling asleep (sleep onset), time of waking up (sleep offset), time of getting out of bed (out-of-bed time), number of awakenings during the night, total duration of awakenings, sleep inertia, mood upon awakening and subjective quality of sleep, and latency to getting up (latency to getting up). Based on these parameters, total sleep time, sleep efficiency, and time in bed were calculated [37].

Every participant received MotionWatch 8 actigraph (CamNtech, Fenstanton, UK), which was worn on the wrist of non-dominant hand. The actigraphy allowed the collection of the following data: time of going to bed, time of lights out (lights-out time), time of falling asleep (sleep onset), time of waking up (sleep offset), time of getting out of bed (out-of-bed-time), total sleep time, time in bed, number of awakenings during the night, sleep latency, latency of getting out of bed (latency to getting up), sleep efficiency, and Sleep Fragmentation Index (SFI). SFI was calculated based on the formula:

(1) $$SFI=\left(\frac{Mobile\;time\;during\;sleep}{Time\;between\;SOn\;and\;SOff}+\frac{Number\;of\;immobile\;bouts\leq 1\;min}{Number\;of\;total\;immobile\;bouts}\right)\times 100\%$$

Actigraphy was carried out for at least 7 days concurrently with the completion of a sleep diary. The epoch time of 30 s was the duration for which the MotionWatch accumulated samples before storing the result in memory.

### 2.5. Determination of Melatonin and DLMO

Plasma samples with internal standard (IS) were extracted with dichloromethane. After evaporation of the organic layer, the extract was dissolved in 5 mM aqueous ammonium acetate solution and analyzed using liquid chromatography linked with a tandem mass spectrometry (LC-MS/MS) system (LCMS-8030, Shimadzu, Kyoto, Japan) equipped with a C18 2.6 μm, 100 A, 75 mm × 2.1 mm Kinetex column linked to a 2.1 ID UHPLC precolumn (Phenomenex, Torrance, CA, USA). The mobile phase consisted of methanol, a 5 mM aqueous solution of ammonium acetate, and formic acid (40:60:0.1; *v*/*v*/*v*).

DLMO was chosen as the time point at which the MELA concentration exceeds 20 pg/mL; the threshold was chosen based on our previous validation study (Dermanowski et al., 2022) [1].

### 2.6. Statistical Analysis

We hypothesized that DLMO would correlate with scales assessing the severity of insomnia and the severity of daytime sleepiness, as well as chronotype questionnaires and sleep parameters in actigraphic recordings. The Shapiro–Wilk test was used to evaluate normal distribution. To test this hypothesis, Pearson’s correlation coefficients were calculated to determine the relationship between the investigated parameters. Results yielding a *p*-value below 0.05 were considered statistically significant. All calculations and linear analyses were performed using Statistica software (version 13.3, TIBCO Software Inc., Palo Alto, CA, USA).

The power analysis showed that in order to demonstrate the statistical significance of the relationship at the level of r = 0.5, the group size should be at least 20 people.

## 3. Results

### 3.1. Characteristic of Study Group

Twenty-one volunteers participated in the study. DLMO was calculated based on the profile of plasma melatonin concentrations in 12 volunteers (Table 1). In the remaining cases, no time-dependent increase in melatonin concentration was observed or blood could not be collected. The mean DLMO time obtained from the plasma was 21:30 ± 0:45 h (*n* = 12), with a range of 20:24–22:50. The mean results from the actigraphy and sleep diaries are shown in Table 2, and results from clinical scales are presented in Table 3.

The average bedtime was 23:25 ± 1:28 h. Participants turned off the lights (LOT) around midnight. According to the sleep diary, sleep latency (SL) was 14.2 ± 8.5 min, and according to the actigraphy, it was 12.7 ± 6.9 min. Average sleep onset (SOn) was a few minutes after midnight, and sleep offset (SOff) was around 7:30. Total sleep time (TST) was 419.0 ± 37.5 min according to the sleep diary, and 386.8 ± 40.8 min according to the actigraphy. The latency to getting up (LTGU) based on the sleep diary was 12.3 ± 8.3 min and was almost twice as long compared to the actigraphy results (6.9 ± 5.5 min). The Sleep Fragmentation Index (SFI) was 22.6 ± 7.7%. The sleep diaries additionally provide information regarding the latency to lights out (LTLO) which was 33.6 ± 41.6 min. In the subgroup of participants with calculable DLMO, mean LOT and SOn were 25–30 min earlier, and mean BT, SOff, and OOBT were approximately 35–37 min earlier for both the sleep diary and actigraphy (Table 2).

### 3.2. Clinical Scales

**Figure 1:**
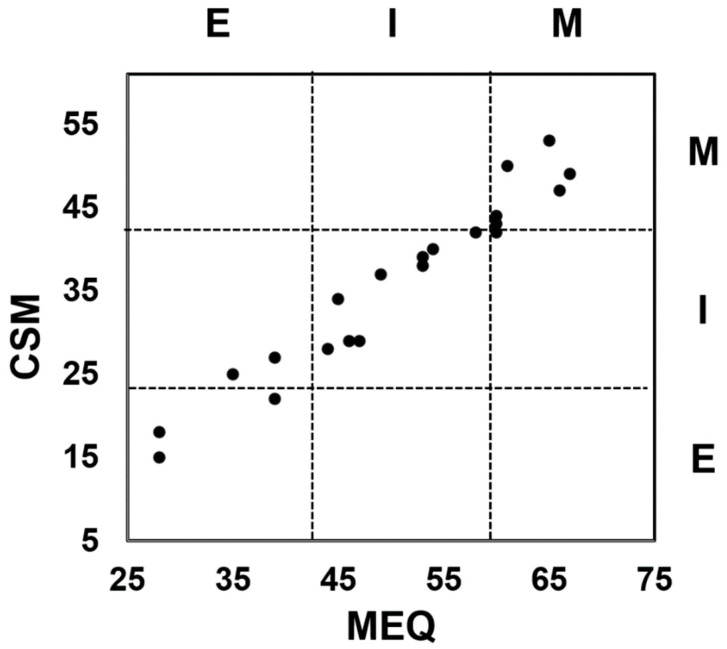
Distribution of chronotype according to Morningness–Eveningness Questionnaire (MEQ) (*x*-axis) and Composite Scale of Morningness (CSM) (*y*-axis). Chronotypes: M = morning, I = intermediate, E = evening.

The score of AIS ≥8 (cut-off value) occurred in six participants (three in the subgroup with calculated DLMO) and ISI ≥ 8 in seven participants (three in the DLMO subgroup). All participants with an AIS score ≥8 also had an ISI score ≥8. A result indicating an excessive daytime sleepiness (ESS ≥ 11) occurred in four (two in the DLMO subgroup) participants. In addition, all subjects with ESS ≥11 score also had an ISI ≥8 score, and three (two in the DLMO subgroup) of them had an AIS ≥8 score (Table 3). The distribution of chronotypes according to MEQ sores was as follows: five (two in the DLMO subgroup) evening chronotypes, nine (four in the DLMO subgroup) intermediate chronotypes, and seven (six in the DLMO subgroup) morning chronotypes. The distribution of chronotypes according to the CSM questionnaire among the study participants was as follows: 3 (1 in the DLMO subgroup) evening chronotypes, 12 (6 in the DLMO subgroup) intermediate chronotypes, and 6 (5 in the DLMO subgroup) morning chronotypes (Figure 1).

### 3.3. Relationships of DLMO with Sleep Parameters and Clinical Scales

DLMO significantly correlated with the Insomnia Severity Scale (r = 0.60, *p* = 0.038) and Epworth Sleepiness Scale (r = 0.61, *p* = 0.034). The correlation coefficient between the DLMO and the Athens Insomnia Scale was also high, however, insignificant (r = 0.57, *p* = 0.054). There were no significant correlations between DLMO and chronotype scales MEQ and CSM (Table 4).

Surprisingly, DLMO was not correlated with sleep onset and sleep offset. However, DLMO was strongly associated with SFI (r = 0.67, *p* = 0.017), but with no other actigraphic parameter. DLMO significantly correlated with the LTLO (r = −0.64, *p* = 0.034). No other significant correlations were found between DLMO and other sleep diary parameters (Table 4).

### 3.4. Correlations between Sleep Parameters and Clinical Scales

AIS was strongly associated with ISI (r = 0.92, *p* < 0.001) and ESS (r = 0.75, *p* < 0.001), and there was also a low correlation found with SE (A) (r = −0.44, *p* < 0.05). ISI and ESS correlated significantly with each other (r = 0.78, *p* < 0.001) and both with sleep onset (r = 0.45, r = 0.48, respectively, *p* < 0.05). Both chronotype scales, MEQ and CSM, strongly correlated with each other (r = 0.97, *p* < 0.001), with LOT from sleep diary and actigraphy (r = −0.65 to −0.78), as well as with OOBT from sleep diary (r = −0.81, r = −0.83, respectively). Also, BT (D) was associated (*p* < 0.05) with ESS (r = 0.45), MEQ (r = −0.67), and CSM (r = −0.65).

### 3.5. Other Correlations

We observed a negative relationship between age and TST (both from actigraphy and sleep diary). SFI positively correlated with age (r = 0.50, *p* < 0.05) and negatively with SE (A) (r = −0.50, *p* < 0.05). The other demographic variables did not correlate with sleep parameters.

## 4. Discussion

To the best of our knowledge, this is the first study to determine the association between DLMO and validated clinical scales, i.e., Athens Insomnia Scale (AIS), Insomnia Severity Index (ISI), Epworth Sleepiness Scale (ESS), and one of the few studies evaluating such an association with chronotype questionnaires Morningness–Eveningness Questionnaire (MEQ) and Composite Scale of Morningness (CSM), as well as with a broad set of sleep and circadian rhythm parameters on a population of healthy adults.

The main finding of this study is a significant correlation between DLMO and clinical scales assessing insomnia (ISI) and daytime symptoms of poor night-time sleep (ESS) and a lack of correlation between DLMO and sleep parameters such as sleep onset and sleep offset (measured by sleep diary and actigraphy). Additional findings include a positive correlation between DLMO and Sleep Fragmentation Index (SFI) (measured by an actigraphy) and a negative correlation between total sleep time (TST) and age. This study did not reveal a significant correlation between DLMO and chronotype questionnaires (MEQ and CSM).

The results of our study did not confirm the association between DLMO and sleep onset and sleep offset. Previously published studies have indicated significant correlations between sleep parameters and DLMO [19,20,21]. A clear strong correlation was especially observed in one of these studies [19]. In the above study, the participants were healthy young adults who sleep at their normal times, with no restrictions on their sleep schedules. Melatonin samples were collected at home and participants naturally have day-to-day variations in their times of sleep. This represents a major difference from our study, where participants were older and in most cases their daily rhythm time was fixed by their work schedule (they worked at least 40 h per week). Moreover, in our study, melatonin samples were collected under laboratory conditions, but sleep parameters were collected at home. Thus, one of the possible explanations for the lack of correlation between DLMO and sleep onset and sleep offset found in this study could be linked to more fixed schedule of daily rhythm.

Participants in this study appear to have largely aligned their sleep rhythms with socio-occupational demands rather than their biological rhythms. Similar observation were described in the paper of Burgess and Eastman (2005) [22]. In assessing the associations between sleep parameters and DLMO, the authors found weaker correlations of DLMO with sleep onset and sleep offset in a group of participants with a fixed sleep pattern compared to a group of free sleepers (ad libitum, sleep when they want). The authors of the above paper conclude that DLMO can be easily estimated only in individuals whose sleep is minimally disturbed by environmental factors, and that further work is needed to determine whether DLMO can be determined in individuals with strong social and occupational influences on sleep. Similar conclusion supports a recent study on insomniacs showing a weak correlation between DLMO and sleep onset and sleep offset in a group with a fixed schedule of bedtime [38]. The study indicates that DLMO is strongly related with sleep onset and sleep offset only in cases of unaffected sleep rhythm.

Both DLMO and sleep parameters from the sleep diary and actigraphy correlate with scales of insomnia and daytime sleepiness (ISI, ESS). At the same time, ISI and ESS scales simultaneously correlated with time to fall asleep (sleep onset). This is the first study to identify a correlation between DLMO and the above scales, and there are only a few studies evaluating the associations between these scales and sleep parameters in healthy volunteers. The only study identified on this topic evaluated the relationship between DLMO and insomnia scales in people with shift work disorder (SWD) [17]. The control group consisted of asymptomatic shift workers in whom DLMO was 04:42 ± 3:25 h, compared to 22:42 ± 2:21 h in the SWD group. This indicates impaired adaptation of melatonin secretion in the SWD group and normal adaptation to the inverted circadian rhythm in the control group.

Another explanation for the lack of correlation between DLMO and sleep parameters in our study might also be the discrepancy between the study conditions under which DLMO was measured (<50 lux), and the conditions under which clinical data were collected. Sleep diaries and actigraphy were conducted under real-world conditions where light intensity was not controlled (e.g., electronic devices with blue screens). It has been shown that DLMO occurs earlier in most individuals under home conditions than under laboratory conditions, and melatonin secretion is reduced and delayed (suppression by light) under home conditions, compared to laboratory conditions [39]. Additionally, it is known that a room light of >200 lux significantly suppresses melatonin secretion and delays DLMO [40], which in real-world conditions can significantly affect the time to fall asleep. Hence, we concluded that the relation between DLMO and sleep parameters determined under strictly controlled conditions may be less pronounced than under uncontrolled home conditions.

The results of this study also revealed a moderate correlation between DLMO and the Sleep Fragmentation Index (SFI) (r = 0.67). SFI is an important parameter in assessing sleep quality and depends primarily on the number of awakenings during sleep. A higher number of awakenings during sleep is associated with sleep disturbance, and an elevated SFI translates into lower sleep efficiency [41,42]. A significant but low correlation between DLMO and SFI was also observed in a study in diabetic patients (r = 0.35) [43]. It seems that a later sleep rhythm may be associated with poorer sleep quality, as well as higher SFI.

Another finding is a significant negative correlation between TST and age, confirming that the total sleep time (TST) decreases with age. This result is consistent with observations reported in the literature, and a decrease in sleep time with age has been reported in previous studies [44,45]. Interestingly, we found that the Sleep Fragmentation Index (SFI) increases with age. This primarily indicates increasing difficulty in maintaining sleep with age, which may be related to the deficit in melatonin secretion [46,47].

The only link of DLMO with sleep diary data found in this study is a negative correlation between DLMO and LTLO, thus later DLMO was associated with shorter latency to lights out. This finding is somewhat interesting, however, requires further observation.

In regard to the MEQ and CSM questionnaires, our study found no correlation between these questionnaires and DLMO, with both chronotype scales very strongly correlating with each other (r = 0.97). In another study, on patients with delayed sleep–wake phase disorder (DSWPD), a strong correlation (r = −0.70) was found between DLMO and MEQ. However, in other studies on healthy volunteers, the association between DLMO and MEQ was much weaker (r = −0.25/−0.35) [18,21,48]. A recently published meta-analysis on the topic found that DLMO was correlated with MEQ score; however, of the 29 studies included in the analysis, a non-significant association was found in 14 data sets, and the total R^2^ was only 0.147 [13]. The reason probably lies in the high variability of MEQ; we still do not know what individual factors affect the MEQ score. There is no doubt that MEQ is only slightly related to DLMO. It should also be kept in mind that most of the studies for which the relationship between DLMO and chronotype have been studied have been conducted in young people aged 20–29 years. Our study was conducted on the older age group, with the average age of 36 years. It should also be pointed out that the participants in our study had a low variation in their chronotypes falling within the intermediate type chronotype. Thus, the variability in MEQ and CSM may have been too small to reveal a significant correlation between the DLMO and MEQ and CSM.

The study has several important limitations. The biggest limitation is the relatively small group of people in whom the DLMO has been successfully determined. This makes the statistical power of the analyses performed to be very low, and it is possible that even moderate or strong correlations may not have achieved statistical significance. Another limitation is the high percentage of people in whom DLMO could not be determined (9/21). It is likely that a higher sampling rate and a longer sampling period, or a sampling period that takes into account bedtime, could improve this result. A certain limitation of the study was the artificial laboratory environment for measuring DLMO, detached from the actual habits and behaviours of the subjects. It is possible that melatonin taken under home conditions would have correlated better with the actual circadian rhythm of the study participants. In the future, it would be reasonable to conduct a study on a larger group of volunteers and take into account such factors as fixed vs. free sleep rhythm and laboratory conditions vs. natural conditions of sampling. In order to improve the determinability of DLMO, the sampling time could be adjusted to the individual sleep pattern or the sampling time should be elongated.

## 5. Conclusions

DLMO correlates with clinical scales assessing insomnia and daytime sleepiness (ISI and ESS), but not with behavioural parameters such as sleep onset and sleep offset, derived from a sleep diary and actigraphy, or chronotype (assessed by MEQ and CSM questionnaires). In addition, later DLMO is associated with poorer sleep quality, symptoms of insomnia (according to the ISI) and daytime sleepiness (according to the ESS), as well as difficulty in maintaining sleep as measured by the Sleep Fragmentation Index (SFI). This demonstrates that there is a growing problem, even in healthy individuals, probably associated with a lifestyle disconnected from the biological circadian rhythm. The results of this study also indicated that since DLMO is not correlated with behavioural parameters in healthy adults, it cannot be used as a substitute for sleep parameters derived from a sleep diary or actigraphy. It therefore seems reasonable to use both methods simultaneously.

## Figures and Tables

**Table 1:** Characteristics of the study group.

	All Participants (*n* = 21)	Participants with Calculated DLMO (*n* = 12)
Age in years	36.2 ± 7.9 (26–54)	36.2 ± 8.8 (26–54)
Sex	10 M/11 F	6 M/6 F
Working hours/week	44.9 ± 8.7 (38–72)	42.9 ± 4.5 (40–50)

**Table 2:** Mean values of actigraphic/sleep diary parameters, expressed as mean ± SD.

	All Participants		Participants with Calculated DLMO	
	Sleep Diary (*n* = 20) *	Actigraphy (*n* = 21)	Sleep Diary (*n* = 11) *	Actigraphy (*n* = 12)
BT	23:25 ± 1:28 h	-	22:50 ± 0:50 h	-
LTLO	33.6 ± 41.6 min	-	22.7 ± 11.6 min	-
LOT	23:52 ± 1:17 h	23:50 ± 1:05 h	23:23 ± 0:38 h	23:24 ± 0:39 h
SL	14.2 ± 8.5 min	12.7 ± 6.9 min	13.9 ± 11.0 min	13.6 ± 7.0 min
SOn	00:06 ± 1:18 h	00:03 ± 1:05 h	23:36 ± 0:43 h	23:38 ± 0:42 h
SOff	07:36 ± 1:21 h	07:30 ± 1:23 h	07:01 ± 0:49 h	06:54 ± 0:48 h
TST	419.0 ± 37.5 min	386.8 ± 40.8 min	419.5 ± 38.9 min	372.8 ± 38.5 min
SE	87.4 ± 5.8%	84.0 ± 3.8%	88.0 ± 6.2%	82.4 ± 3.8%
LTGU	12.3 ± 8.3 min	6.9 ± 5.5 min	9.6 ± 5.5 min	7.3 ± 5.8 min
OOBT	07:48 ± 1:28 h	07:37 ± 1:23 h	07:11 ± 0:51 h	07:01 ± 0:50 h
SFI	-	22.6 ± 7.7%	-	25.0 ± 8.7%

Abbreviations: BT = bedtime, LTLO = latency to lights out, LOT = lights-out time, SL = sleep latency, SOn = sleep onset, SOff = sleep offset, TST = total sleep time, SE = sleep efficiency, LTGU = latency to getting up, OOBT = out-of-bed time, SFI = Sleep Fragmentation Index, * one of the participants did not return the sleep diary.

**Table 3:** Mean values of clinical scale scores: mean ± SD.

Clinical Scales				
All Participants (*n* = 21)			Participants with Calculated DLMO (*n* = 12)	
Mean Scores		Range	Mean Scores	Range
AIS	6.1 ± 3.7	1–13	6.7 ± 4.1	1–13
ISI	5.6 ± 5.2	0–16	5.6 ± 5.1	0–16
ESS	8.0 ± 5.3	0–22	7.4 ± 6.3	0–22
MEQ	50.3 ± 11.9	28–67	55.9 ± 9.5	49–67
CSM	35.8 ± 10.8	15–50	41.0 ± 9.1	22–49

AIS = Athens Insomnia Scale, ISI = Insomnia Severity Index, ESS = Epworth Sleepiness Scale, MEQ = Morningness–Eveningness Questionnaire, CSM = Composite Scale of Morningness.

**Table 4:** Correlations between DLMO and sleep parameters calculated from sleep diary (D), actigraphy (A), and clinical scales.

	Correlation with DLMO r Coefficient
Age	0.45
BT (D)	0.29
LOT (D)	0.03
OOBT (D)	−0.03
TST (D)	−0.09
SL (D)	0.15
SE (D)	0.17
LTLO (D)	−0.64 *
LTGU (D)	0.03
LOT (A)	−0.08
SOn (A)	−0.06
SOff (A)	−0.10
TST (A)	−0.13
SL (A)	0.01
SE (A)	−0.32
SFI (A)	0.67 *
AIS	0.57
ISI	0.60 *
ESS	0.61 *
MEQ	−0.19
CSM	−0.11

Abbreviations: DLMO = dim light melatonin onset, BT = bedtime, LOT = lights-out time, OOBT = out-of-bed time, TST = total sleep time, SL = sleep latency, LTLO = latency to lights out, LTGU = latency to getting up, SOn = sleep onset, SOff = sleep offset, SFI = Sleep Fragmentation Index, AIS = Athens Insomnia Scale, ISI = Insomnia Severity Index, ESS = Epworth Sleepiness Scale, MEQ = Morningness–Eveningness Questionnaire, CSM = Composite Scale of Morningness, (D) = sleep diary parameter, (A) = actigraphic parameter. * *p* < 0.05. Note: DLMO vs. actigraphy/scales with *n* = 12, DLMO vs. sleep diary with *n* = 11.

## Data Availability

The data presented in this study are available on request from the corresponding author. All relevant data from the study are presented in this manuscript or published [1].
